# Exploration of Novel Inhibitors for Bruton’s Tyrosine Kinase by 3D QSAR Modeling and Molecular Dynamics Simulation

**DOI:** 10.1371/journal.pone.0147190

**Published:** 2016-01-19

**Authors:** Rohit Bavi, Raj Kumar, Light Choi, Keun Woo Lee

**Affiliations:** Division of Applied Life Science (BK21 Plus Program), Systems and Synthetic Agrobiotech Center (SSAC), Plant Molecular Biology and Biotechnology Research Center (PMBBRC), Research Institute of Natural Science (RINS), Gyeongsang National University (GNU), 501 Jinju-daero, Jinju, 52828 Republic of Korea; Institute of Molecular Genetics IMG-CNR, ITALY

## Abstract

Bruton’s tyrosine kinase (BTK) is a cytoplasmic, non-receptor tyrosine kinase which is expressed in most of the hematopoietic cells and plays an important role in many cellular signaling pathways. B cell malignancies are dependent on BCR signaling, thus making BTK an efficient therapeutic target. Over the last few years, significant efforts have been made in order to develop BTK inhibitors to treat B-cell malignancies, and autoimmunity or allergy/hypersensitivity but limited success has been achieved. Here in this study, 3D QSAR pharmacophore models were generated for Btk based on known IC_50_ values and experimental energy scores with extensive validations. The five features pharmacophore model, Hypo1, includes one hydrogen bond acceptor lipid, one hydrogen bond donor, and three hydrophobic features, which has the highest correlation coefficient (0.98), cost difference (112.87), and low RMS (1.68). It was further validated by the Fisher’s randomization method and test set. The well validated Hypo1 was used as a 3D query to search novel Btk inhibitors with different chemical scaffold using high throughput virtual screening technique. The screened compounds were further sorted by applying ADMET properties, Lipinski’s rule of five and molecular docking studies to refine the retrieved hits. Furthermore, molecular dynamic simulation was employed to study the stability of docked conformation and to investigate the binding interactions in detail. Several important hydrogen bonds with Btk were revealed, which includes the gatekeeper residues Glu475 and Met 477 at the hinge region. Overall, this study suggests that the proposed hits may be more effective inhibitors for cancer and autoimmune therapy.

## Introduction

Bruton’s tyrosine kinase (BTK) is a cytoplasmic, non-receptor tyrosine kinase from a Tec-family kinase, which is expressed in most of the hematopoietic cells and plays an important role in many cellular signaling pathways [1–4]. In the life cycle of B-lineage cells BTK plays a central role in proliferation, development, differentiation, survival and apoptosis [5]. BTK is characterized by five structural domains including N-terminal pleckstrin homology (PH) domain, a proline-rich TEC homology (TH) domain, Src homology 3 (SH3) followed by Src homology 2 (SH2) domain and a C-terminal kinase domain (BTK-KD). The PH domain plays an essential role in the regulation and functioning of the BTK. The PH domain contains the site for binding the transcription factors (BAP-135/TFII-I), inhibitors (PIN 1, 1BTK) [6] and activators (phosphatidylinositol 3,4,5-trisphosphates and G-protein βγ) [7]. The TH domain is stretch of 80 amino acid residues having a conserved region for zinc cofactor binding site and proline-rich segment [8], which serves as a binding site for protein kinase C-beta (PKC-β) [9]. Initially BTK is activated by phosphorylating Tyr551 in the activation loop of C-terminal kinase domain; however further activation occurs in the SH3 domains, were autophosphorylation of Tyr223 occurs [10, 11].

In the lymphoid lineage, Btk is only expressed in B cells and is not found in natural killer or T cells. B cells play a significant role in the pathogenesis of several autoimmune diseases. Clinical studies have shown that depletion of mature B cells can be efficacious in multiple sclerosis, systemic lupus erythematosus (SLE), and rheumatoid arthritis (RA) [12]. Even though Btk is expressed in the myeloid cell lineage, mutations in the Btk gene lead to prominent B cell—specific defects in mice and humans, hence it has been considered as a target for the selective inhibition of B cells [13]. In humans, mutations in the BTK gene is characterized by a B-lymphocyte developmental defect, giving rise to a primary immunodeficiency disease called X-linked agammaglobulinemia (XLA). The individuals suffering from XLA is characterized by lack of circulating B lymphocytes, therefore unable to generate immunoglobulins, and thus cannot stand humoral immune responses. Similarly, mutation in the mouse-Btk gene results in X-linked immunodeficiency (xid), a related but less severe phenotype than XLA [14–18]. B cell expansion and the production of autoantibodies by polyclonal B cell activation is a characteristic of RA [19], thus selective inhibition of Btk may be an attractive therapeutic target for B cell inhibition in RA as well as for B cell lymphoma.

Ibrutinib (PCI-32765), Dasatinib, LFM-A13, CC-292, and ONO-WG-307 are well known Btk inhibitors, with varying specificities [20]. For example, LFM-A13 and Dasatinib not only inhibits Btk with an IC_50_ value of 2.5 μ*M* and 5 *nM*, but also binds to other kinases such as PLK3, JAK2 and SRC family members (HCK, SRC, CSK) [21–24]. Also, Ibrutinib (PCI-32765) interferes with B-cell functioning and leads to hypogammaglobulinemia [25]. Though many inhibitors are reported and few are in clinical trials, none are FDA approved and are selective to Btk. Hence, designing potent and specific Btk inhibitors becomes crucial.

Here, we used computer-aided drug design approaches to identify potent and novel inhibitors which can cause inhibition of Btk. A 3D QSAR pharmacophore model was built from the chemical features present in already known inhibitors. The best model, Hypo 1, was validated and used for database screening. The potential compounds were filtered by checking their drug like properties. Binding conformations of the selected hit compounds were predicted by molecular docking studies. Finally, the appropriate binding modes of final hit compounds were revealed by molecular dynamics (MD) simulations and free energy calculation studies.

## Materials and Methods

### Collection of dataset

**Fig 1:**
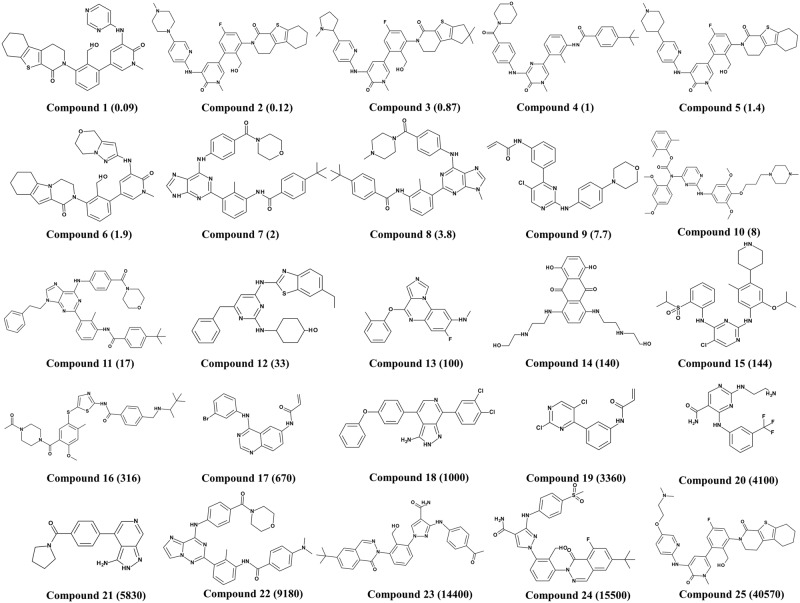
2D Chemical structures of 25 Btk inhibitors in the training set used for hypothesis generation along with their IC_50_ values.

To perform pharmacophore modeling calculations a dataset of 85 known inhibitors of Btk with diverse scaffold collected from different literature resources [26–29] and classified into two different data sets: (i) a training set and (ii) a test set. Training set was used to generate the hypothesis while the generated hypothesis was validated by a test set. Among these 85 compounds, 25 were selected as the training set compounds (Fig 1) based on their IC_50_ values and structural diversity. The remaining 60 compounds were used as a test set for validating the hypothesis. The inhibitory activity value of these compounds was between 0.09 nmol/L to 40570 nmol/L. The data set compounds were classified into active (IC_50_ < 100 nmol/L, +++), moderately active (100 nmol/L ≤ IC_50_ < 10000 nmol/L, ++) and inactive (IC_50_ ≥ 10000 nmol/L, +) based on their IC_50_ value. ChemSketch was used to sketch the 2D form of all the data set compounds (ACD Inc., Toronto, Canada) and was consequently exported to Discovery Studio v3.5 (DS) for their corresponding 3D structure generation.

### Generation of pharmacophore model

Before performing the pharmacophore modeling, *Feature Mapping* protocol was used to identify the chemical features of the training set compounds that are important in inhibition of Btk. The chemical features identified by the feature mapping protocol were used to generate pharmacophore models using the 3D QSAR Pharmacophore Generation protocol available in DS by correlating the experimental activities values of compounds with their chemical structures. BEST algorithm was used to generate low energy conformation of the compounds. Uncertainty value was set to 3 while other parameters had default values. Debnath method was used to identify and evaluate the top ten hypotheses based on the activity values offered by the training set compounds [30]. Debnath method suggests that the model having a high correlation coefficient, the lowest total cost, the lowest RMS deviation, and the total cost close to the fixed cost and far from the null cost is considered as the best quantitative hypothesis [30]. The reliability of hypothesis depends on the difference between the total cost of the generated hypothesis and the null hypothesis.

### Pharmacophore validation

The best hypothesis selected from the top ten hypotheses was subjected to validation by Fisher’s randomization and the external test set method. The statistical significance of the model was computed by employing Fischer’s randomization method [31]. This method is used to verify that the selected hypothesis is not generated by chance and also to checks if there is a strong correlation between the biological activities and the chemical structures. The confidence level was set to 95% and nineteen random spreadsheets were generated [32]. This was done by randomizing the activity of these compounds by using the same features and parameters used to generate the original pharmacophore hypothesis. During this process if any of the random pharmacophore hypotheses showed better statistical values than Hypo1, then the Hypo1 was generated by random correlation [33]. Test set was used to determine whether Hypo1 can predict and classify the compounds correctly in their activity scale of molecules other than the training set compounds. External test set contained 60 chemically diverse compounds with wide range of inhibitory activity values when compared to the training-set compounds. The selected pharmacophore hypothesis (Hypo1) was used to predict the activity values of test-set compounds. The predicted and experimental activity values were plotted to observe the range of correlation between them.

### Virtual screening and drug-likeness prediction

Virtual screening of chemical databases is done to identify new scaffolds that can trigger or inhibit the activity of a particular target. The benefit of virtual screening is that the hit compounds can be gained easily for biological testing as compared to de novo design methods [34]. In this work, Hypo1 was used as a 3D structural query in virtual screening to retrieve a novel lead compound for Btk inhibition from four different chemical databases: Chembridge, NCI, Asinex, and Maybridge. The screened compounds that mapped all the features of Hypo1 were selected as hit compounds. Estimated activity values, geometric fit values ADME properties and Lipinski’s rule of five were used as a filter for further refinement of mapped compounds. During ADME investigation the compounds were checked for low blood—brain barrier (BBB), optimal solubility, good absorption, non-inhibition to CYP2D6 and non-hepatotoxicity; if the molecule had values of 3, 3, and 0 for BBB, solubility, and absorption, respectively, it was considered that the molecule had good solubility, absorption, and BBB [35]. Lipinski’s rule of five [36] estimates the absorption and intestinal permeability of a compound. Lipinski’s rule states that, the compounds that are well absorbed have a logP value less than 5, less than 5 hydrogen-bond donors, less than 10 hydrogen-bond acceptors, molecular weight of less than 500, and fewer than ten rotatable bonds. The compounds having better estimated activity values and filtered by drug-like properties was conceded further for molecular docking.

### Molecular docking

In the drug designing process, molecular docking is used as a filtering method, as it is used to find the most appropriate conformation and interactions of each hit compound at the active site of protein. Docking studies were performed using GOLD program version 5.2.2. For molecular docking calculation a high resolution (1.80 Å) crystal structure (PDB code 3OCS) of Btk bound with an inhibitor was selected as protein molecule [37]. The water molecules and hetero atoms were removed from protein. CHARMm force field was used to add hydrogen atoms to the protein molecule. The binding site was identified based on the volume occupied by the co-crystallized ligand in the protein. The hit compounds along with training set compounds were docked into the active site of protein. ND1H protonation state was kept for all the histidine tautomers as observed in the crystal structure. To predict the binding affinity of the ligand to the target protein, Gold fitness score function was used as the default scoring function while rescoring was done using Chemscore. Based on the scoring functions (high Goldscore and low Chemscore), molecular interactions, and the formation of hydrogen bonds between the ligand and the active site residues of protein the best docked poses were selected.

### Molecular dynamics simulations

The molecular dynamic (MD) simulations of Btk in complex with the final hit compounds obtained from docking studies and the most active compound from the training set were performed using GROMACS 4.5.7 package with CHARMm27 force field [38]. Topology files for ligands were generated by SwissParam [39]. The system were solvated in a dodecahedron box containing TIP3P water model to form an aqueous environment and neutralized with Na+ counter ions. 10000 minimization steps were carried out with steepest descent algorithm to remove possible bad contacts from initial structures until tolerance of 2000 kJ/mol. The energy minimized system was then subjected to equilibration in three different steps. A constant temperature controlled by V-rescale thermostat [40] was applied for 100 ps at 300k in the first phase of equilibration. Later, 100 ps NPT ensemble was applied at 1 bar of pressure followed by 20 ns of production run under the same ensembles. During this process, Parrinello-Rahman barostat was used to maintain the pressure of the system [41]. In the equilibration process the solvent molecules with counter ions were allowed to move while protein backbone was restrained. SETTLE and LINCS algorithm were used to constrain the geometry of water molecules and bond involving hydrogen atoms respectively [42, 43]. Periodic boundary conditions were applied to avoid edge effects. Particle Mesh Ewald (PME) algorithm were applied to calculate the long range electrostatic interactions [44]. A cut off distance of 9Å and 10Å was set for Coulombic and van der Waals interactions. Each simulation was run for 20 ns and the coordinate data was stored at every picosecond (ps). All the analysis of MD simulations was carried out by VMD [45] and DS software.

### Binding free energy calculations

The binding free energy calculations were performed using Molecular Mechanics/Poisson-Boltzmann Surface Area (MM/PBSA) method as described previously [46, 47]. For calculating binding free energy 40 snapshots of protein-ligand complex were selected evenly from 0 to 20 ns of MD trajectories as per earlier studies [46, 48]. Different energy parameters have been calculated using MM/PBSA method by using the same snapshots [46, 48– 49]. The binding interaction between protein and ligand was calculated in three terms such as solvation contribution (ΔEsol), van der Waals contribution (ΔEvdw) and the electrostatic contribution (ΔEele).

## Results and Discussion

### Pharmacophore modeling

**Fig 2:**
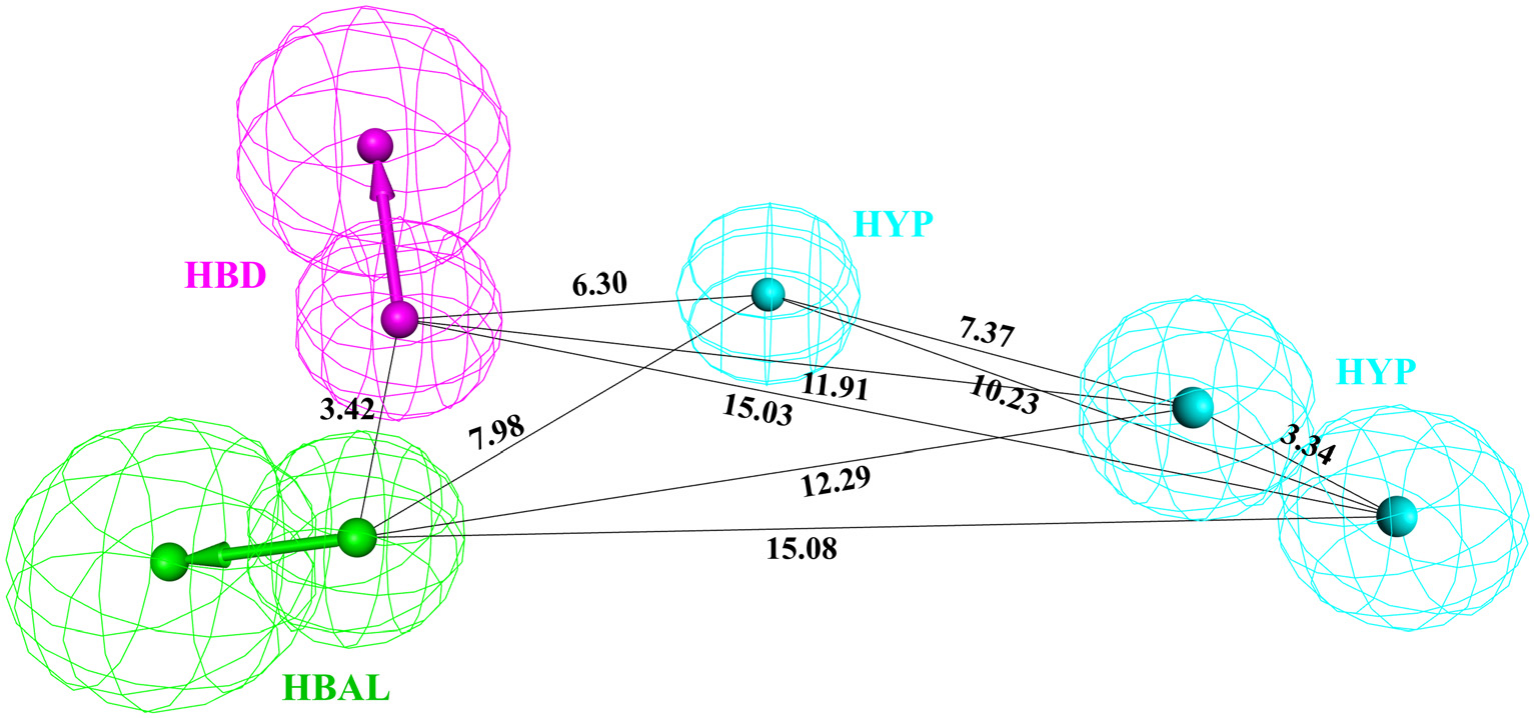
Chemical features of the best pharmacophore 'Hypo 1' with its distance constraints. 'Hypo 1' consists of one hydrogen bond acceptor lipid (HBAL: Green), one hydrogen bond donor (HBD: Magenta), three hydrophobic (HYP: Cyan) features.

HypoGen algorithm was used to build the quantitative hypotheses by correlating the estimated and the experimental activity values of the Btk inhibitors. The hypothesis was generated by using the training set of 25 chemically diverse compounds (Fig 1) with activity values ranging from 0.09 nmol/L to 40570 nmol/L by selecting hydrogen bond acceptor lipid (HBAL), hydrogen bond donor (HBD), hydrophobic (HYP), hydrogen bond acceptor (HBA), and ring aromatic (RA) features as suggested by *Feature Mapping protocol*. A total of 10 hypotheses were generated each having five chemical features. Hypo1 is the representative hypothesis, showing a good geometric spatial arrangement consisting five chemical features namely 1 HBAL, 1 HBD, and 3 HYP (Fig 2). Hypo1 fulfilled all the statistical parameters such as the configuration cost of 12.21; total cost (125.42) which was close to the fixed cost (116.43) and away from the null cost (238.29) indicates that Hypo1 was the best hypothesis. There was a high correlation coefficient of 0.981 along with large cost difference of 112.87 and lowest RMS value of 0.68 (Table 1). By considering all the above parameters it was revealed that the statistical values of Hypo1 was best as compared to the other hypothetical structures. As a result, Hypo1, was selected as the best hypothesis for further analysis (Fig 2). The 3D spatial relationship and distance constraint of Hypo1 is depicted in Fig 2.

**Table 1:** Statistical data of ten pharmacophore hypotheses generated by HypoGen.

Hypo No.	Total Cost	Cost Difference^a^	RMSD^b^	Correlation (R^2^)	Max Fit	Features^c^
Hypo 1	125.42	112.87	0.68	0.981	11.68	1 HBAL, 1 HBD, 3 HYP
Hypo 2	122.71	115.58	0.84	0.970	10.97	1 HBA, 1 RA, 3 HYP
Hypo 3	122.07	116.22	0.87	0.968	11.19	1 HBAL, 3 HYP, 1 RA
Hypo 4	121.84	116.45	0.88	0.967	10.78	1 HBD, 1 HBA, 3 HYP
Hypo 5	119.49	118.8	0.95	0.962	11.97	1 HBAL, 1 HBD, 3 HYP
Hypo 6	118.64	119.65	1.02	0.956	10.83	1 HBAL, 3 HYP, 1 RA
Hypo 7	118.45	119.84	1.03	0.955	10.47	1 HBA, 3 HYP, 1 RA
Hypo 8	117.74	120.55	1.05	0.953	10.86	1 HBAL, 3HYP, 1 RA
Hypo 9	117.38	120.91	1.06	0.953	11.25	1 HBAL, 1 HBD, 3 HYP
Hypo 10	117.33	120.96	0.99	0.959	10.44	2 HBA, 1 HYP, 1 RA

^a^ Cost difference, difference between the null cost and the total cost. The null cost of ten scored hypotheses is 238.29, the fixed cost value is 106.43. All costs are represented in bit units.

^b^ RMSD: deviation of the log (estimated activities) from the log (experimental activities) normalized by the log (Uncertainties).

^c^ HBAL, hydrogen bond acceptor lipid; HBD, hydrogen bond donor; HYP hydrophobic; HBA, hydrogen bond acceptor; and RA, ring aromatic.

To elucidate the predictive accuracy of Hypo1 the training set compounds were classified into active (IC_50_ < 100 nmol/L, +++), moderately active (100 nmol/L ≤ IC_50_ < 10000 nmol/L, ++) and inactive (IC_50_ ≥ 10000 nmol/L, +) based on their IC_50_ value. Regression analysis was used to estimate the activity of each compound. Hypo1 estimated the inhibitory activity value in the same order of magnitude for all the training set compounds (Table 2) except two moderately active and two inactive compounds which were overestimated as active and moderately active compounds, respectively.

**Table 2:** Experimental and estimated activity of training set compounds based on Hypo 1.

Compound No.	Fit Value	Exp IC_50_ nmol/L	Pred IC_50_ nmol/L	Error^a^	Exp Scale^b^	Pred Scale^b^
1	11.16	0.09	0.22	+2.4	+++	+++
2	11.33	0.12	0.15	+1.3	+++	+++
3	10.57	0.87	0.87	-1	+++	+++
4	10.07	1	2.8	+2.8	+++	+++
5	10.33	1.4	1.5	+1.1	+++	+++
6	9.76	1.9	5.5	+2.9	+++	+++
7	10.39	2	1.3	-1.5	+++	+++
8	10.01	3.8	3.2	-1.2	+++	+++
9	9.78	7.7	5.3	-1.5	+++	+++
10	9.43	8	12	+1.5	+++	+++
11	9.45	17	12	-1.5	+++	+++
12	8.79	33	52	+1.6	+++	+++
13	8.63	100	76	-1.3	+++	+++
14	8.93	140	38	-3.7	++	+++
15	8.40	142	130	-1.1	++	++
16	7.91	320	390	+1.2	++	++
17	6.98	670	3400	+5	++	++
18	7.97	1000	350	-2.9	++	++
19	7.27	3400	1700	-1.9	++	++
20	7.48	4100	1100	-3.9	++	++
21	6.92	5800	3800	-1.5	++	++
22	6.33	9200	15000	+1.7	++	+++
23	6.54	14000	9200	-1.6	+	++
24	6.57	15000	8600	-1.8	+	++
25	5.40	41000	130000	+3.2	+	+

^a^ Error, ratio of the predicted activity (Pred IC_50_) to the experimental activity (Exp IC_50_) or its negative inverse if the ratio is <1.

^b^ Activity scale: IC_50_ < 100 nmol/L = +++ (active), 100 nmol/L ≤ IC_50_ < 10000 nmol/L = ++ (moderate active), IC_50_ ≥ 10000 nmol/L = + (inactive).

**Fig 3:**
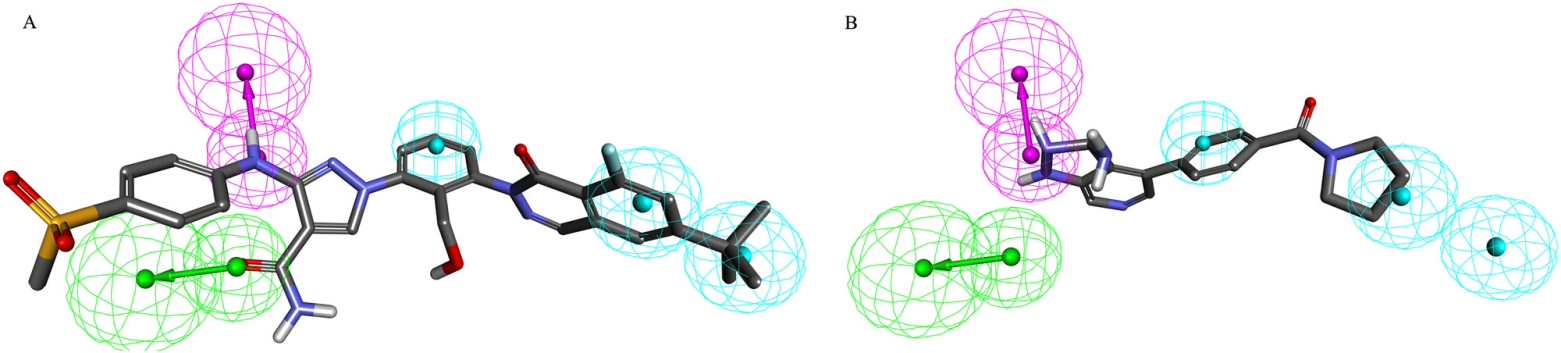
The best pharmacophore model Hypo1 aligned to training set compounds: A) most active compound 1 (IC50 0.09 nmol/L) and B) least activity compound 20 (IC50 40570 nmol/L). The most active compound mapped to all four features in Hypo 1, whereas the least active compound mapped only two features.

Hypo 1 aligned with the most active (IC_50_ = 0.09 nmol/L) compound and the least active (IC_50_ = 40570 nmol/L) compound in the training set as depicted in Fig 3. Clearly, all the hypothetical features were perfectly mapped by the most active compound (Fig 3A), whereas the least active compound (Fig 3B) failed to fit on one HBA and one HYP feature. This reveals the difference in activities among the most active and the least active compounds. This analysis suggests that Hypo1 was able to differentiate the compounds based on the activity values with high accuracy (Table 2). Hypo1 was further validated using the test-set and Fischer randomization method.

### Pharmacophore validation

#### Test set validation

**Fig 4:**
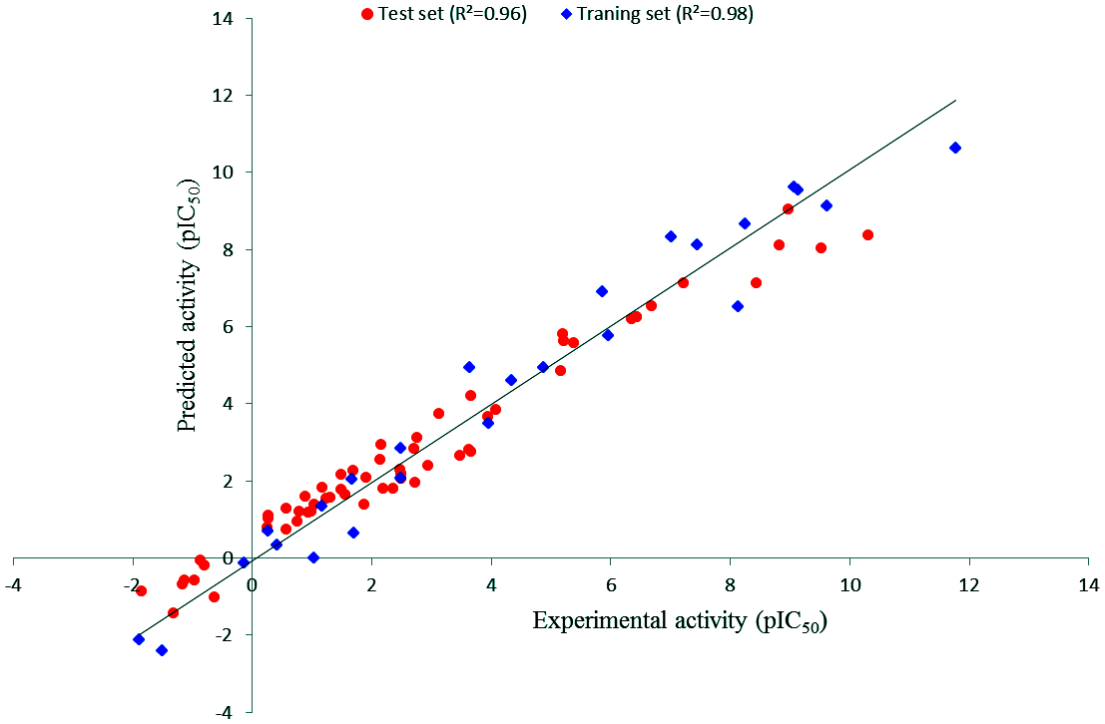
Correlation plot between Hypo1 predicted Btk inhibitory activities and experimental activities of 60 test set compounds and 25 training set compound.

A good pharmacophore should have the ability to predict and classify the compounds according to their activities scale. Hypo1 was validated by external validation (test set) process which consist of 60 structurally diverse compounds other than the training set compounds (S1 Table) and were classified into active (IC_50_ < 100 nmol/L, +++), moderately active (100 nmol/L ≤ IC_50_ < 10000 nmol/L, ++) and inactive (IC_50_ ≥ 10000 nmol/L, +) respectively. One moderately active compound was underestimated as being inactive, and two inactive molecules were overestimated as moderately active compounds. The remaining compounds were classified correctly, indicating that Hypo1 was able to predict the activities of compounds in their own activity scales as depicted in Table 3. The linear regression between the Hypo1-predicted activities and experimental inhibitory activities of the test-set compounds showed a correlation coefficient (r) value of 0.96 (Fig 4). This result shows the predictive capacity of Hypo1 to discriminate between the active and moderately active compounds.

**Table 3:** Evaluation of estimated and experimental activity (IC_50_) values of test set compounds using Hypo 1.

Compound number	Fit Value	Experimental IC_50_ (nmol/L)	Predicted IC_50_ (nmol/L)	Error^a^	Experimental Scale^b^	Predicted Scale^b^
1	11.31	0.15	0.43	+0.36	+++	+++
2	11.08	0.26	0.24	-1.10	+++	+++
3	11.01	0.30	0.51	+0.60	+++	+++
4	10.99	0.32	0.56	+0.57	+++	+++
5	10.92	0.37	0.57	+0.66	+++	+++
6	10.88	0.41	0.95	+0.43	+++	+++
7	10.85	0.44	0.83	+0.53	+++	+++
8	10.78	0.52	0.36	-1.45	+++	+++
9	10.40	1.27	2.2	+0.58	+++	+++
10	10.39	1.29	3	+0.43	+++	+++
11	10.39	1.29	2.8	+0.46	+++	+++
12	10.26	1.74	3.6	+0.48	+++	+++
13	10.26	1.74	2.1	+0.83	+++	+++
14	10.18	2.10	2.6	+0.80	+++	+++
15	10.16	2.17	3.4	+0.64	+++	+++
16	10.12	2.41	5	+0.48	+++	+++
17	10.10	2.51	3.3	+0.76	+++	+++
18	10.07	2.68	3.4	+0.78	+++	+++
19	10.06	2.78	4	+0.69	+++	+++
20	10.00	3.17	6.2	+0.51	+++	+++
21	9.97	3.43	4.7	+0.73	+++	+++
22	9.94	3.65	4.9	+0.74	+++	+++
23	9.86	4.37	6	+0.72	+++	+++
24	9.86	4.41	8.7	+0.50	+++	+++
25	9.83	4.70	5.2	+0.90	+++	+++
26	9.77	5.38	9.8	+0.54	+++	+++
27	9.69	6.47	4.0	-1.61	+++	+++
28	9.68	6.67	8.1	+0.82	+++	+++
29	9.58	8.41	13.0	+0.64	+++	+++
30	9.57	8.60	19.0	+0.45	+++	+++
31	9.55	8.90	6.1	-1.45	+++	+++
32	9.48	10.52	6.12	-1.72	+++	+++
33	9.43	11.78	10.1	-1.16	+++	+++
34	9.42	12.06	8.0	-1.50	+++	+++
35	9.42	12.06	9.0	-1.34	+++	+++
36	9.33	14.80	17.1	+0.86	+++	+++
37	9.32	15.21	17.1	+2.14	+++	+++
38	9.30	15.76	22.9	+0.68	+++	+++
39	9.23	18.75	11.04	-1.69	+++	+++
40	9.15	22.64	43.0	+0.52	+++	+++
41	8.99	32.22	14.28	-2.25	+++	+++
42	8.93	37.14	16.6	-2.23	+++	+++
43	8.91	38.61	67.0	+0.57	+++	+++
44	8.91	38.71	16.6	-2.41	+++	+++
45	8.79	50.90	39.35	-1.29	+++	+++
46	8.73	58.77	47.0	-1.25	+++	+++
47	8.26	174.33	129.9	-1.34	++	++
48	8.25	178.33	333.06	+0.53	++	++
49	8.24	181.87	280.83	+0.64	++	++
50	8.17	215.87	267.74	+0.80	++	++
51	7.75	570.61	3700	+0.15	++	++
52	7.71	620.37	518	-1.19	++	++
53	7.71	620.87	287	-2.16	++	++
54	7.60	798.80	3142	+0.25	++	++
55	7.37	1359.21	1255	-1.08	++	++
56	6.84	4587.11	1270	-3.61	++	++
57	6.67	6787.21	3330	-2.03	++	++
58	6.61	7821.13	12700	+0.61	++	+
59	6.37	13667.60	1050	-13.01	+	++
60	6.03	29655.90	2687	-11.03	+	++

^a^ Error, ratio of the predicted activity to the experimental activity or its negative inverse if the ratio is <1.

^b^ Activity scale: IC_50_ < 100 nmol/L = +++ (active), 100 nmol/L ≤ IC_50_ < 10000 nmol/L = ++ (moderate active), IC_50_ ≥ 10000 nmol/L = + (inactive).

#### Fischer’s randomization method

**Fig 5:**
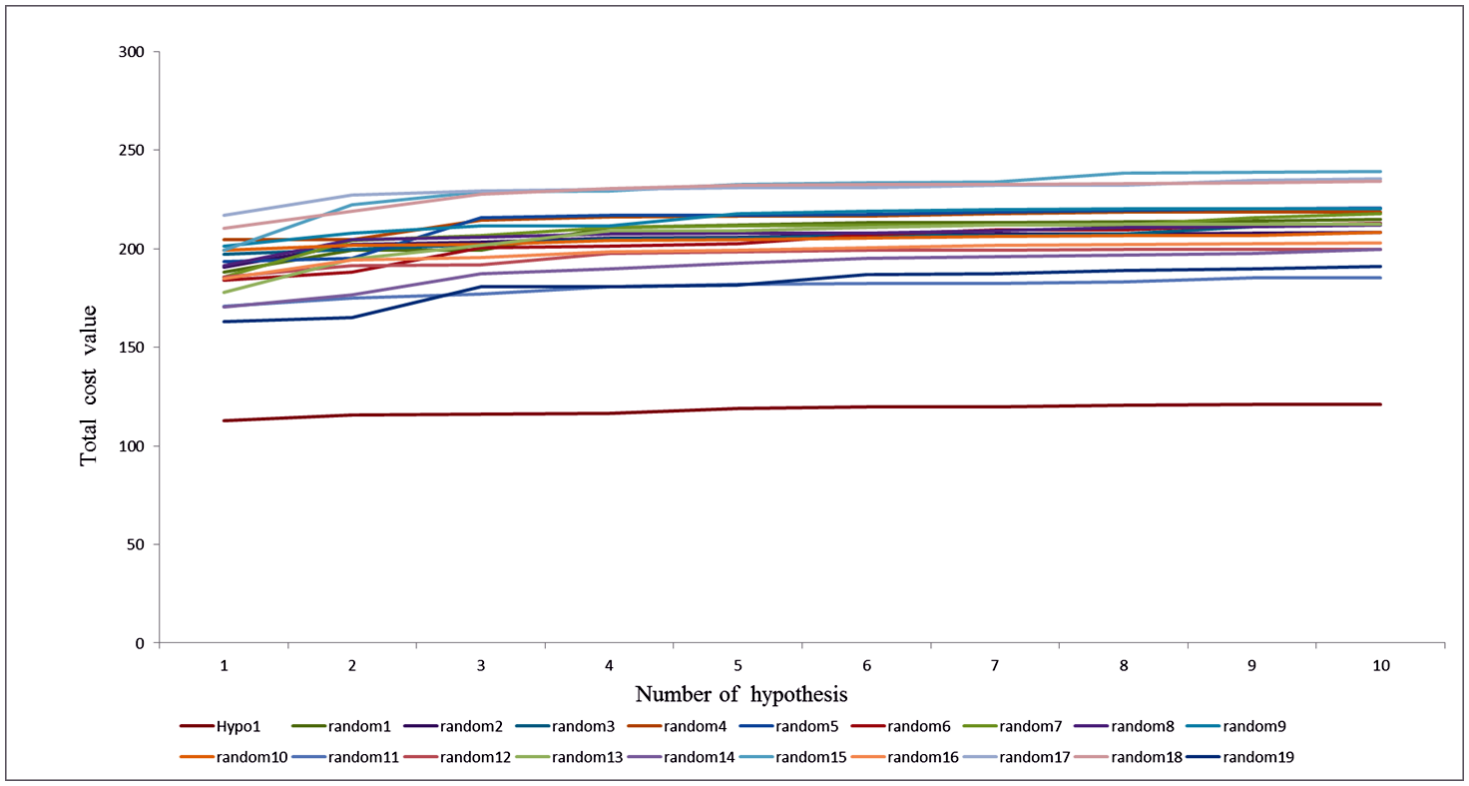
Comparison between the total cost of Hypo1 with the total costs of the 19 scrambled runs generated during the Fisher randomization run.

To estimate the statistical relevance of Hypo1 Fischer’s test was applied. Here we set a 95% confidence level; as a result 19 random spreadsheets were generated by arbitrarily reassigning the experimental activity values to each compound in the training set, and a hypothesis was created for each spreadsheet (Fig 5). The formula used to calculate the significance of the hypothesis is S = [1–(1+X)/Y]×100, where X denotes total number of hypotheses with total cost that are lower than the original hypothesis, and Y represents the total number of HypoGen runs (initial+random runs). Here, X = 0 and Y = (1+19), hence 95% = {1–[(1+0)/(19+1)]}×100. The generated random spreadsheets showed least total cost value for Hypo1 as compared to other hypothesis, which indicates that Hypo1 is far more superior to all other random hypotheses and was not generated by chance.

### Virtual screening

In the drug discovery process, virtual screening of chemical databases is an alternative method to the high-throughput screening technique. Chemical features of Hypo1 play an important role in mapping and screening out novel compounds from a database. We therefore used Hypo1 as a 3D structural query to screen Asinex, Chembridge, Maybridge, and NCI databases which contains 213262, 50000, 59652 and 238819 compounds, respectively. Among these, Hypo 1 mapped 29249 compounds that had all the chemical features of Hypo1. Further these compounds were filtered down to 3723 by applying a filter of maximum fit value greater than 10. However, even if a molecule passes various filters, it may not be active towards Btk, hence we tested the filtered compounds for their ADME properties and Lipinski’s rule of five. ADME and Lipinski's rule of five plays an important role in sorting the chemical compounds based on drug-like properties. Therefore, ADME and Lipinski's rule of five were used as a filter to sort these molecules. Finally, a total of 23 compounds satisfied the drug-like properties and were subjected to molecular docking to study their critical interactions with the important amino acids present in the active site of Btk.

### Molecular docking

**Fig 6:**
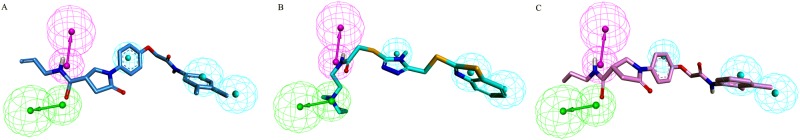
Hypo1 mapped onto the hit compounds. A) Hit 1, (B) Hit 2, (C) Hit 3. The HBAL, HBD and HYP features are displayed in green magenta and cyan, respectively.

The training set compounds along with 23 drug-like hits resulted from pharmacophore modeling were subjected to docking using GOLD program so as to refine the retrieved hit compounds and to eliminate the false positives. To gauge the accuracy of GOLD and to examine the parameters to produce the appropriate binding orientation the co-crystal was docked in the active site of Btk. It resulted in an acceptable RMSD value of 1.04 Å between the predicted structure and the co-crystal (S1 Fig). Therefore, by using the same parameters the candidate compounds were docked. The GOLD fitness scores, molecular interactions with the binding site residues, binding modes, and Chemscore were considered as key components in selecting the best conformation of the docked compounds. GOLD fitness score differentiates molecules based on their interacting ability. GOLD fitness score value greater than that of most active compound was taken as cut-off for the further screening of compounds. Chemscore estimates the total free energy change that occurs upon ligand binding and was used as the rescoring function. The most active compound in the training set has scored a GOLD fitness score of 69.7 and Chemscore of -30.7 (Table 4). Thus, the compounds were selected based on GOLD fitness score greater than 69.7, Chemscore lower than -30.7, and the ligand conformations satisfying the necessary interactions in the active site. Finally, three hit compounds fulfilled the above criteria and also mapped well to the pharmacophoric features of Hypo 1 (Fig 6) were characterized as final hits.

**Table 4:** Comparison of Gold fitness score, Chemscore and average binding energy of Btk and reference inhibitor/hit1/hit2/hit3 complex.

Systems	Gold fitness score	ChemScore	Average binding energy (KJ/mol)
BTK + Inhibitor	69.7	-30.7	-84.1
BTK + Hit 1	72.4	-29.3	-87.9
BTK + Hit 2	71.7	-36.9	-81.3
BTK + Hit 3	70.6	-36.1	-92.0

### Molecular dynamics simulations

**Fig 7:**
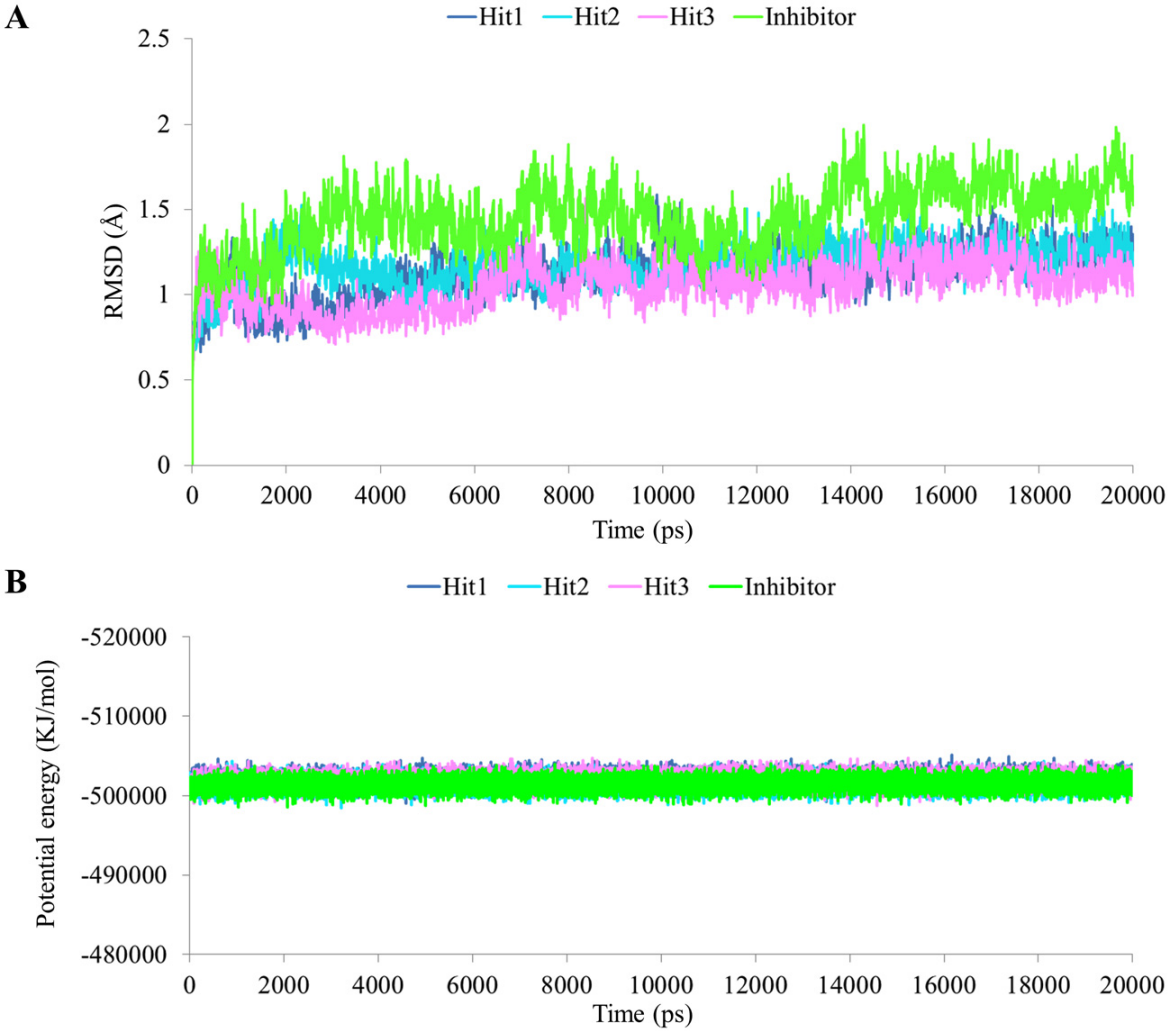
The RMSD and potential energy graph for four complex systems. (A) The RMSD profile for the backbone atoms of Btk protein. (B) The potential energy of the system. These graphs were calculated during 20 ns MD simulations for each complex. Blue, cyan, pink, and green lines represent Hit1, Hit2, Hit3, and Inhibitor respectively.

**Fig 8:**
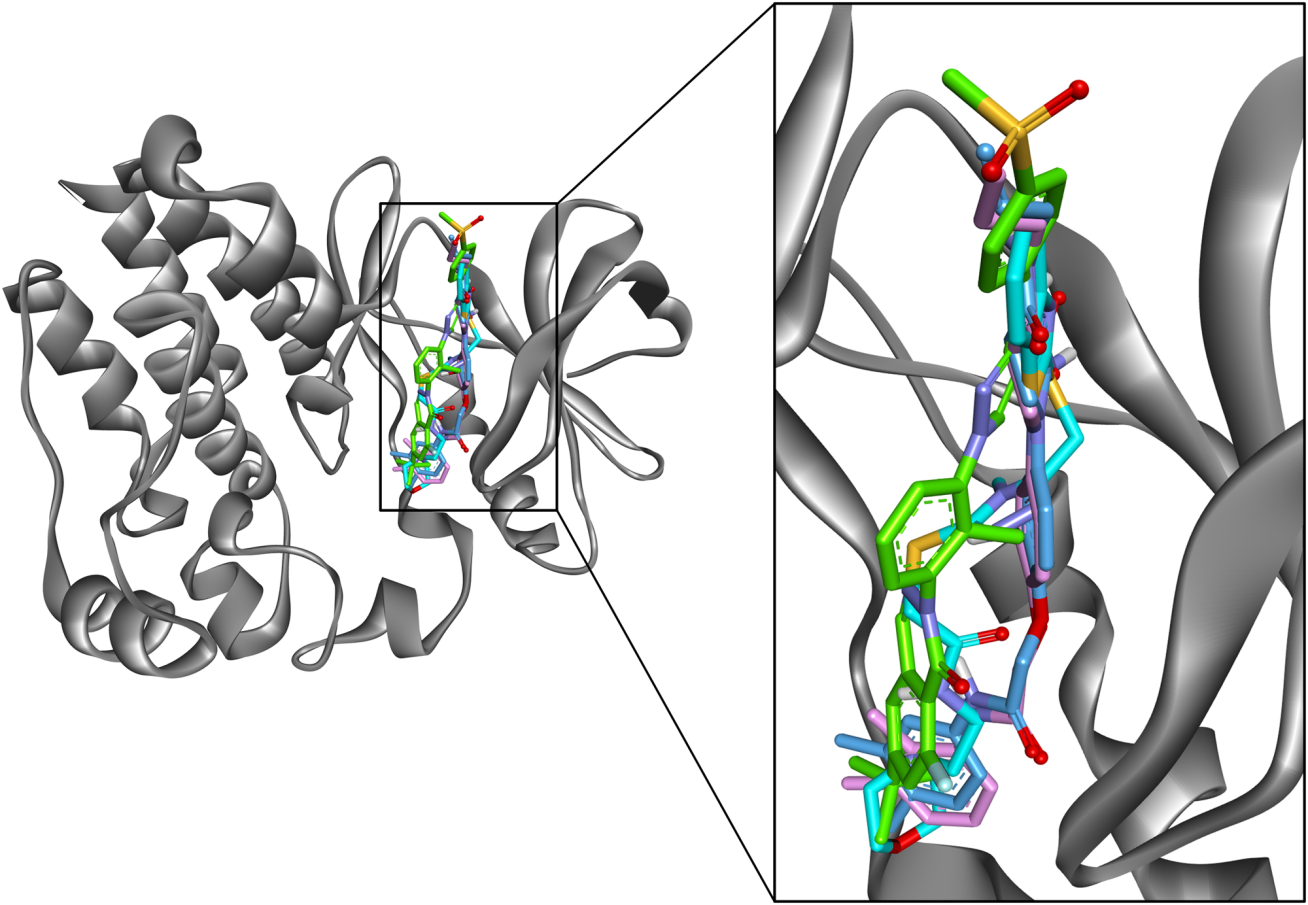
The binding mode of the three hit compounds and reference inhibitor in the active site of Btk. All compounds in their representative structures were superimposed (left) and enlarged (right). The Btk protein is shown in gray color solid ribbon while the compounds are depicted by sticks. Blue, cyan, pink, and green sticks represent Hit1, Hit2, Hit3, and Inhibitor respectively.

**Fig 9:**
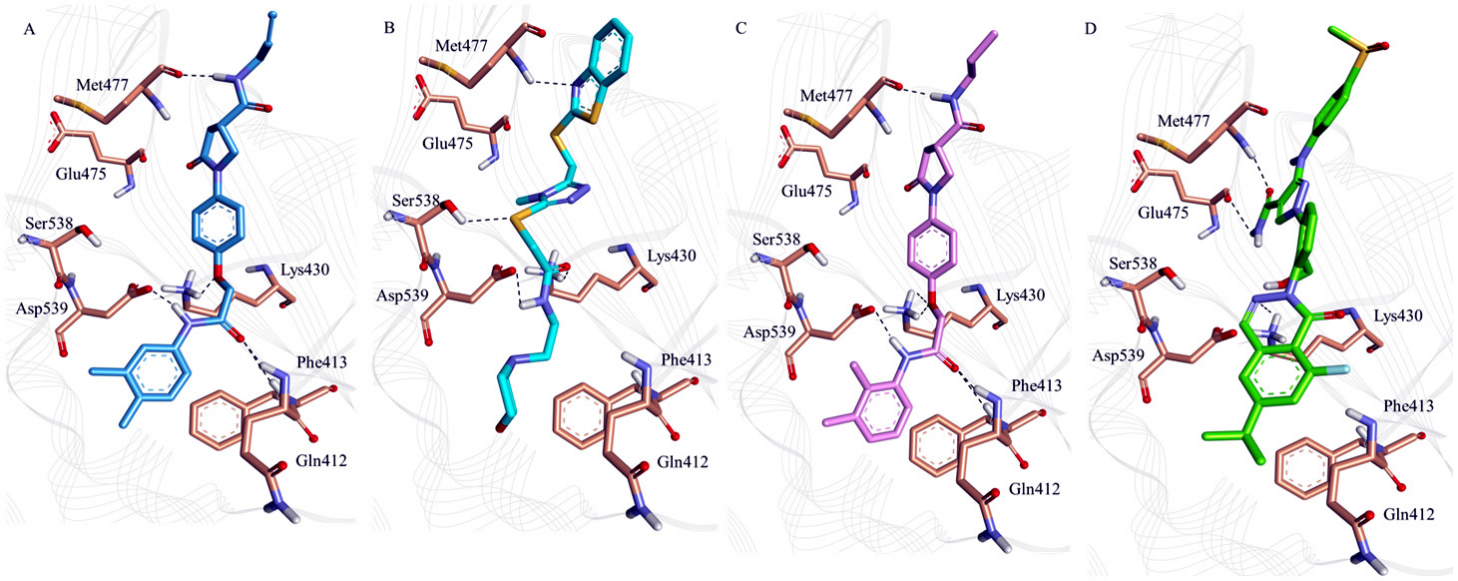
The binding conformation and hydrogen bonding interactions of the three hit compounds and reference inhibitor in the active site of Btk. (A) Hit1: blue (B) Hit2: cyan (C) Hit3: pink and (D) Inhibitor: Green. Hydrogen bond interactions between proteins and compounds are shown as black dotted line. Only polar hydrogen atoms are shown for clarity.

In order to further validate the results and to predict more reliable ligand—receptor interaction MD simulations were performed. The 20 ns MD simulations were done to understand the conformational changes and dynamic behavior with each other by taking the best docked conformation of three hits and a reference compound as the initial structure. All the four systems were subjected to the MD simulation. To explore the dynamic stability of the complexes during simulation the root mean square deviation (RMSD) of protein backbone atoms (Fig 7A) and potential energy (Fig 7B) of the system were calculated. The RMSD values observed for the complexes were in between 0.8 Å to 1.7 Å throughout the simulations which shows that system are well converged. The average RMSD values obtained during simulation for hit1, hit2, hit3, and inhibitor were 1.12 Å, 1.17 Å, 1.05 Å, and 1.45 Å respectively. The potential energy of the system was stable throughout the simulation indicating that no abnormal behavior occurred in the protein. The last 5 ns trajectories were used to analyze the binding mode of the representative structures of four systems. When all the representative structures were superimposed, it was found that the binding pattern of hit compounds was similar to reference compound (Fig 8). The substrate binding pocket of Btk was formed by Gln412, Phe413, Lys430, Glu475, Met 477, Ser538 and Asp539 amino acids. These key residues were also found to interact with the reference inhibitor and hit compounds. In case of Hit compounds, Hit1 formed hydrogen bond interactions with Gln412, Phe413, Lys430, Met477, Asp539 and hydrophobic interaction with Leu408, Gly411, Ala428, Ala478, Gly480, Asp521, Leu528, Leu542, Ser543, Tyr551 (Fig 9A, Table 5). The benzene moiety of hit1 was involved in π -π and σ-π interaction with Phe413 and Val416, respectively. In hit2 binding, hydrogen bonds with Lys430, Met477, Ser538 and Asp539 were observed (Fig 9B, Table 5). Hit2 showed hydrophobic interactions with Leu408, Gln412, Phe413, Val416, Ala428, Met477, Ala478, Gly480, Asn526, Leu528, Ser538, Asp539, Leu542, Ser543, and Tyr551. The benzene moiety of hit2 was involved in π -π interaction with Tyr476 while π-cation interaction with Lys406, and Lys430, respectively. Hit3 formed hydrogen bond interactions with Gln412, Phe413, Lys430, Met477, and Asp539 (Fig 9C, Table 5). Hit3 showed interactions with hydrophobic pocket residues such as Leu408, Gly411, Val416, Ala428, Tyr476, Ala478, Asn479, Gly480, Asn526, Leu528, Leu542, Ser543, Val546, and Tyr551. On the other hand reference compound, inhibitor formed hydrogen bonds with Lys430, Glu475, and Met 477 (Fig 9D, Table 5). Furthermore, Inhibitor was stacked on Lys430 via cation-π interaction. Inhibitor showed hydrophobic interactions with Leu408, Gly411, Gln412, Phe413, Val416, Ala478, Asn479, Gly480, Asn526, Leu528, Leu542, Ser543, Val546, and Tyr551. These results reveals that, the final hit compounds bound to the active site either by forming hydrogen bond interactions, or by σ-π and cation-π interactions and the interacting residues are given in Table 5.

**Table 5:** The molecular interactions between the compounds and Btk protein.

Compound	Hydrogen bond (<3.0 Å)	Hydrophobic interaction	Cation-π interaction	σ-π interaction
Inhibitor	Lys430, Glu475, Met 477	Leu408, Gly411, Gln412, Phe413, Val416, Ala478, Asn479, Gly480, Asn526, Leu528, Leu542, Ser543, Val546, Tyr551	Lys430	
Hit 1	Gln412, Phe413, Lys430, Met 477, Asp539	Leu408, Gly411, Ala428, Ala478, Gly480, Asp521, Leu528, Leu542, Ser543, Tyr551	Phe413	Val416
Hit 2	Lys430, Met 477, Ser538, Asp539	Leu408, Gln412, Phe413, Val416, Ala428, Met477, Ala478, Gly480, Asn526, Leu528, Ser538, Asp539, Leu542, Ser543, Tyr551.	Lys406, Lys430	Tyr476
Hit 3	Gln412, Phe413, Lys430, Met 477, Asp539	Leu408, Gly411, Val416, Ala428, Tyr476, Ala478, Asn479, Gly480, Asn526, Leu528, Leu542, Ser543, Val546, Tyr551		

**Fig 10:**
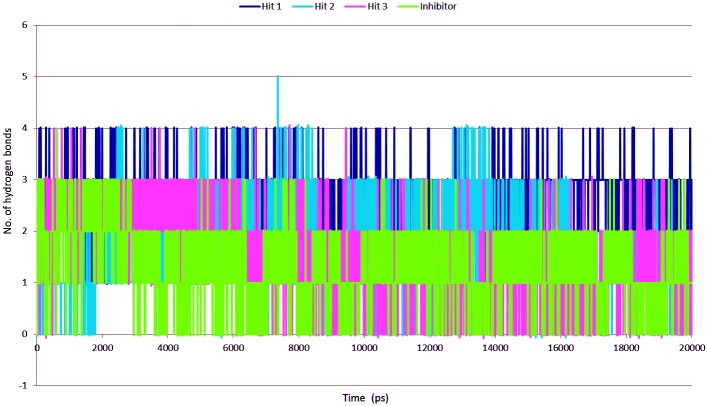
The number of intermolecular hydrogen bonds between protein and compound during 20 ns MD simulations. Blue, cyan, pink, and green colors represent Hit1, Hit2, Hit3, and Inhibitor respectively.

**Fig 11:**
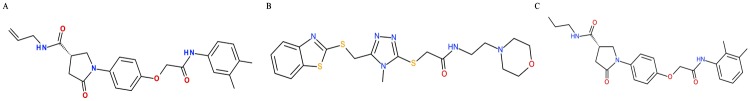
2D structures of the hit compounds.

In order to understand the nature of the binding of drug molecules in the active site, the intermolecular hydrogen bonds between protein and hit compounds were monitored during the simulation period (Fig 10). The average numbers of hydrogen bonds between the Btk protein and hit compounds were 2.6, 1.8, and 1.5 for hit1, hit2, and hit3 respectively. Inhibitor showed almost 1.3 hydrogen bonds throughout the simulation. The reference compound showed relatively less hydrogen bonds than the hit compounds. Further, search by PubChem Structure [50] an online search tool confirmed that these compounds were not tested experimentally for the inhibition of Hck and can be recommended as potential Btk inhibitors. Hence, we suggest that these compounds could be novel as Btk inhibitors (Fig 11).

### Analysis of the binding free energy of Btk and reference inhibitor/hit compounds

**Fig 12:**
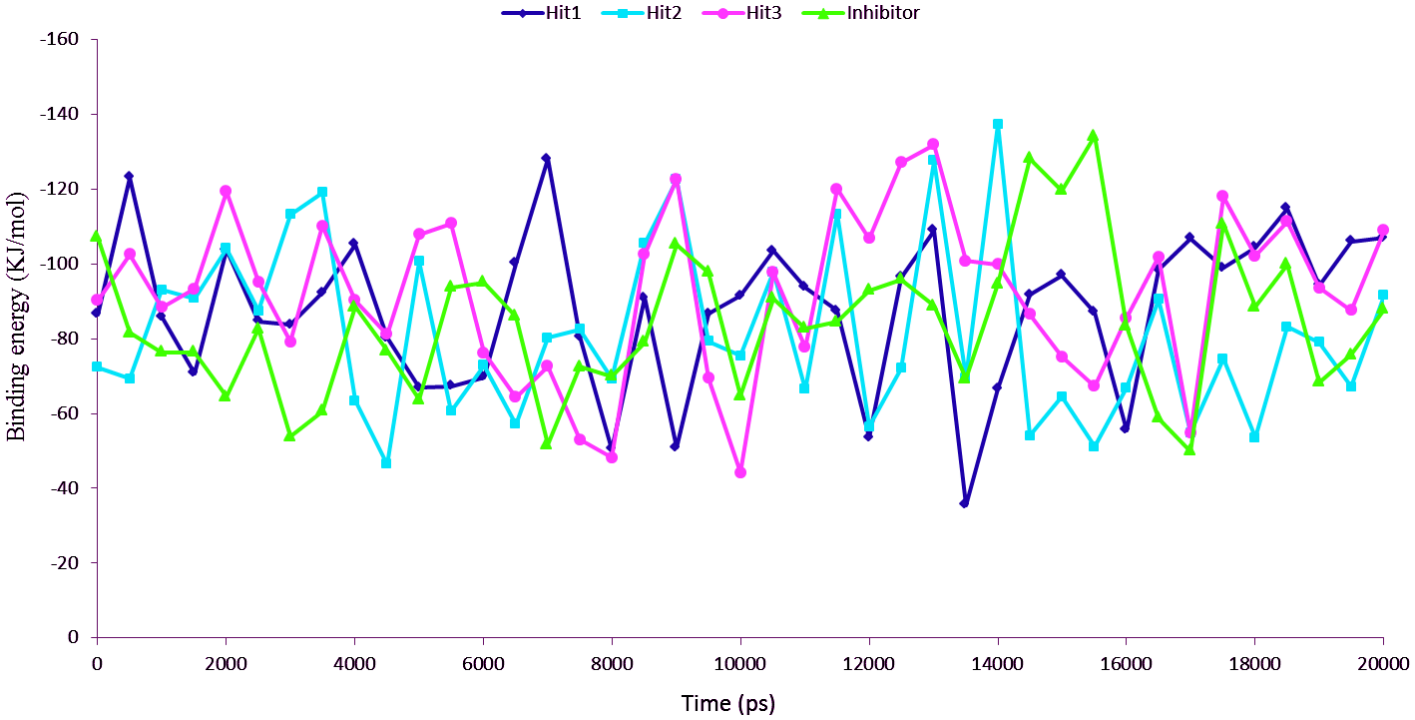
MM/PBSA estimated binding free energy of Btk and hit 1/hit 2/hit 3/ reference inhibitor complex throughout simulation time. Color coding; Hit 1: Blue, Hit 2: cyan, Hit 3: pink and Inhibitor: green.

Calculation of binding energy is a key aspect in understanding the molecular activity of the targeted biomolecules. Estimation of various bonded and non-bonded interactions arbitrating bimolecular association or dissociation offers us supportable information in developing the therapeutic drugs against several biological disorders. MM/PBSA method was used to calculate the binding free energy of each set of protein ligand complex in order to compare the binding affinity between protein with the identified potent lead compounds. The MM/PBSA calculation of Btk-ligand complexes using the reference inhibitor, hit1, hit2 and hit3 as the ligands gave favorable ΔG values in the range of −35 to −137 kJ/mol as depicted in Fig 12. The binding energy showed slight variation in each snapshot as the conformational space was not sampled enough to get converged results. The average binding energy obtained for Btk-ligand complexes were -84.18 kJ/mol (reference inhibitor), -87.96 kJ/mol (hit1), -81.39 (hit2), and -92.09 (hit3) (Table 4). The binding energy obtained from the trajectories produced during the MD simulation, considers the ligand conformation and the fluctuation of the protein in the complex, as a result confirming a proper adjustment of the ligand in the binding site [49, 51]. Btk has charged binding pocket comprising of two Asp, one Glu, two Lys, one Arg, one Gln, and two Asn residues. These amino acid residues form strong ionic interactions with ligands, thus, resulting in strong electrostatic potential in the binding interface of Btk active site. The bound conformation of Btk and ligands shows that ligands get accommodate in the active site of the enzyme through hydrogen bond and hydrophobic interactions.

## Conclusion

Inhibition of Btk has emerged as a new promising target in the field of B cell malignancies and autoimmunity or allergy/hypersensitivity as it is involved in several signaling pathways. Thus as an attempt, a ligand based pharmacophore modeling was done to find the important chemical features which can inhibit the activity of Btk. The five feature pharmacophore model, Hypo1, was developed consisting of 1 HBAL, 1HBD, 3HYP features. Hypo1 had the highest correlation coefficient (0.98), cost difference (112.87), and low RMS (1.68). It was further validated by the Fisher’s randomization method (95%) and test set (r = 0.96). Hence, the best hypothesis Hypo1 was used as a 3D structural query to screen the chemical databases for retrieving new potent inhibitors of Btk. Fit value, Lipinski’s rule of five, and ADMET properties screening assisted us to discard the non-drug-like compounds. Furthermore, the screened drug-like compounds were identified and were subjected to molecular docking study. Finally, molecular dynamic simulation was employed to study the stability of docked conformation and to investigate the binding interaction in details. Several important hydrogen bonds with Btk were revealed, which includes the gatekeeper residues Glu475 and Met 477 at the hinge region. The analyzed results suggested that the binding mode of hit compounds was similar to the reference compounds. The hit compounds bound to the active site residues by forming hydrogen bond, hydrophobic, σ-π and cation-π interactions. Hence, we propose that the final hit compounds as a possible virtual leads to design novel Btk inhibitors.

## Supporting Information

S1 Fig Co-crystal (Gray; PDB ID: 3OCS) overlaped with its docked orientation (yellow). (PNG)

S1 Table Chemical structures of test set compounds with their respective IC_50_ values. (DOC)
