# Supplementary figures and images for: Exploration of Novel Inhibitors for Bruton’s Tyrosine Kinase by 3D QSAR Modeling and Molecular Dynamics Simulation
Source: PLoS One. 2016 Jan 19;11(1):e0147190. doi: 10.1371/journal.pone.0147190 (PMC4718466; doi:10.1371/journal.pone.0147190)

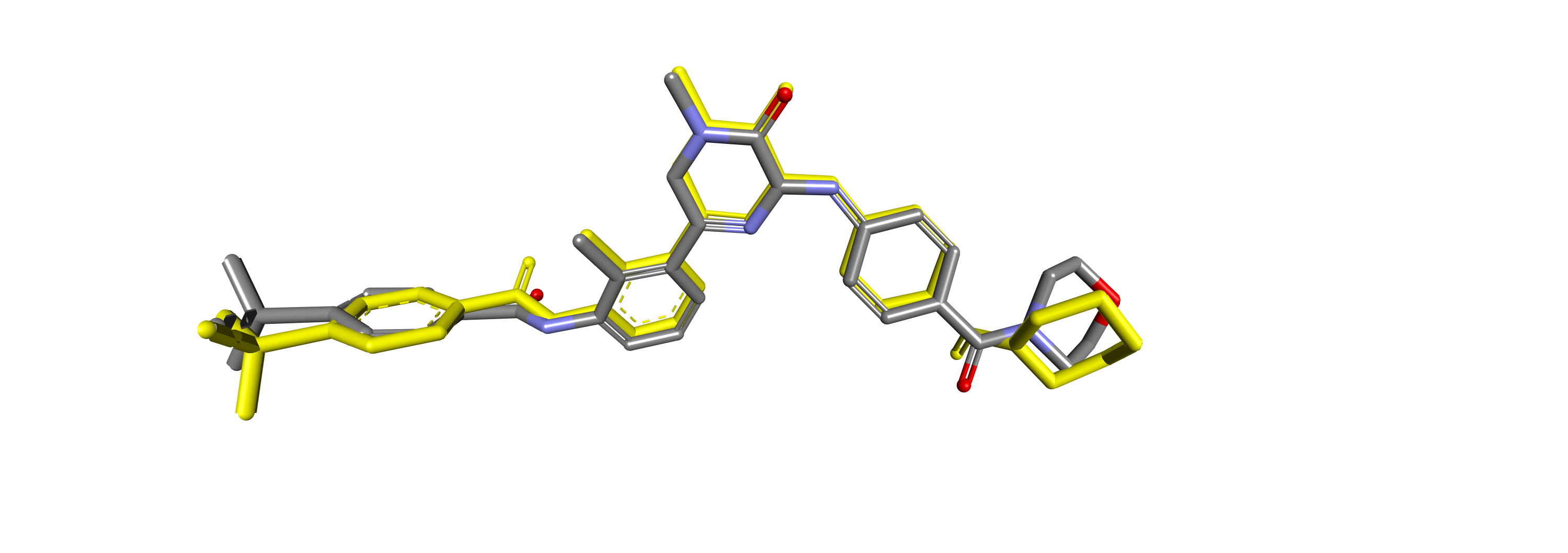

Supplement: S1 Fig — (PNG) [file pone.0147190.s001.png]
