# Supplementary material for: Exploration of Novel Inhibitors for Bruton’s Tyrosine Kinase by 3D QSAR Modeling and Molecular Dynamics Simulation
Source: PLoS One. 2016 Jan 19;11(1):e0147190. doi: 10.1371/journal.pone.0147190 (PMC4718466; doi:10.1371/journal.pone.0147190)
Supplement: S1 Table — (DOC) [file pone.0147190.s002.doc]

**Supplementary Table 1.** Chemical structures of test set compounds with their respective IC_50_ values.

| Compound No. | IC50 (nmol/L) | Structure |
| --- | --- | --- |
| 1 | 0.24 |  |
| 2 | 0.36 |  |
| 3 | 0.43 |  |
| 4 | 0.51 |  |
| 5 | 0.56 |  |
| 6 | 0.57 |  |
| 7 | 0.83 |  |
| 8 | 0.95 |  |
| 9 | 2.1 |  |
| 10 | 2.2 |  |
| 11 | 2.6 |  |
| 12 | 2.8 |  |
| 13 | 3 |  |
| 14 | 3.3 |  |
| 15 | 3.4 |  |
| 16 | 3.4 |  |
| 17 | 3.6 |  |
| 18 | 4 |  |
| 19 | 4 |  |
| 20 | 4.7 |  |
| 21 | 4.9 |  |
| 22 | 5 |  |
| 23 | 5.2 |  |
| 24 | 6 |  |
| 25 | 6.1 |  |
| 26 | 6.1 |  |
| 27 | 6.2 |  |
| 28 | 7.1 |  |
| 29 | 8 |  |
| 30 | 8.1 |  |
| 31 | 8.7 |  |
| 32 | 9 |  |
| 33 | 9.8 |  |
| 34 | 10.1 |  |
| 35 | 11.04 |  |
| 36 | 13 |  |
| 37 | 14.28 |  |
| 38 | 16 |  |
| 39 | 16.6 |  |
| 40 | 17.1 |  |
| 41 | 19 |  |
| 42 | 22.9 |  |
| 43 | 39.35 |  |
| 44 | 43 |  |
| 45 | 47 |  |
| 46 | 67 |  |
| 47 | 129.9 |  |
| 48 | 267.74 |  |
| 49 | 280.83 |  |
| 50 | 287 |  |
| 51 | 333.06 |  |
| 52 | 518 |  |
| 53 | 1050 |  |
| 54 | 1255 |  |
| 55 | 1270 |  |
| 56 | 2687 |  |
| 57 | 3142 |  |
| 58 | 3330 |  |
| 59 | 3700 |  |
| 60 | 12700 |  |
